# Total oxylipin concentrations in NIST SRM 1950 plasma compared to fresh and commercially obtained human plasma

**DOI:** 10.1016/j.jlr.2026.101011

**Published:** 2026-03-02

**Authors:** Nadja Kampschulte, Lilli Scholz, Theresa L. Pedersen, Isabel F. Snodgrass, John W. Newman, Nils Helge Schebb

**Affiliations:** 1 Food Chemistry, School of Mathematics and Natural Sciences, University of Wuppertal, Wuppertal, Germany; 2 EicOsis Human Health, Inc., Davis, CA, USA; 3 West Coast Metabolomics Center, Genome Center, University of California Davis, Davis, CA, USA; 4 Department of Nutrition, University of California Davis, Davis, CA, USA; 5 USDA, ARS, WHNRC, Obesity and Metabolism Research Unit, Davis, CA, USA

**Keywords:** lipidomics, eicosanoids, oxidized phospholipids, epilipodome

## Abstract

Oxylipins are oxidized PUFAs such as hydroxy-PUFAs and epoxy-PUFAs. Total oxylipins are commonly analyzed following base hydrolysis as nonesterified oxylipins by means of LC-MS/MS. For that, a standard operating procedure has been developed, which was successfully tested in an international interlaboratory comparison. Here, we report the concentrations of total oxylipins in National Institute of Standards and Technology (NIST) Standard Reference Material (SRM) 1950 plasma determined by LC-MS/MS analyses in different laboratories, which are similar to those in other freshly prepared human plasma samples. Hydroxy-PUFA of the long-chain PUFA arachidonic acid, DHA, and EPA occur between 1 and 75 nmol/l (0.3–24 ng/ml) and linoleic acid-derived hydroxy-PUFA up to 400 nmol/l (120 ng/ml). The concentrations of arachidonic acid-, DHA-, and EPA-derived epoxy-PUFA range between 2 and 20 nmol/l (0.6–6 ng/ml) and of epoxy-linoleic acid up to 100 nmol/l (32 ng/ml). We also report 70 oxylipins, which cannot be detected (∼<0.5 nmol/l; 0.2 ng/ml), such as multiple hydroxylated PUFA, including so-called specialized proresolving mediators (except for 10,17-dihydroxy-DHA), and most isoprostanes. If the plasma is prepared or stored inappropriately, oxylipins are formed by autoxidation, leading to artificially high levels of total oxylipins. This is shown for the concentrations in different commercially available plasma pools of unknown origin, being 100–1000-fold higher. The concentrations provided here for NIST SRM 1950 plasma and freshly prepared plasma thus inform which concentration range of total oxylipins should be expected in human plasma from healthy subjects and increase the utility of NIST SRM 1950 for analytical quality control protocols.

The majority of oxylipins, i.e. hydroxy-PUFAs and epoxy-PUFAs, in plasma and tissues do not occur free, i.e. non-esterified, but are esterified in phospholipids and other lipids (1, 2, 3, 4, 5, 6).

Several oxylipins are potent lipid mediators, as summarized elsewhere (7, 8, 9). Due to their biological relevance, the baseline levels of nonesterified oxylipins in human plasma—or the expected concentration range—have been described (10, 11, 12, 13, 14, 15) and internationally agreed standards for their quantification have been developed by the scientific community (7).

The biological role of esterified oxylipins is largely unclear. They are determined by the same LC-MS/MS methods following base hydrolysis (saponification) as one of the first steps in sample preparation (16, 17). Two optimized and well-characterized methods for quantification of esterified oxylipins have been described by Quehenberger *et al.* (1) and Ostermann *et al.* (2). For the latter, the effect of plasma preparation and short-term storage, as well as long-term storage at −80°C, has been investigated and was shown to be minor (18, 19). Moreover, the standard operating procedure of the method was validated in an international interlaboratory comparison, yielding consistent results (20).

Here, this standard operating procedure was applied to the National Institute of Standards and Technology (NIST) Standard Reference Material (SRM) 1950—metabolites in frozen human plasma by two different laboratories and to freshly prepared plasma. The data are compared to other studies reporting the concentrations of total oxylipins, allowing to estimate an expected concentration range of oxylipins in human plasma from healthy subjects.

## Materials and methods

For determination of total oxylipins, four aliquots each (technical replicates) of 100 μl of NIST SRM 1950 plasma (NIST, Gaithersburg, MD), commercially available EDTA plasma pool (MaxSpec Reference Plasma, Cayman Chemical, Ann Arbor, MI, #42573, obtained from Biomol, Hamburg, Germany) and fresh EDTA plasma generated from healthy individuals as described (19), approved by the ethics committee of the University of Wuppertal, and in accordance with the guidelines of the Declaration of Helsinki were hydrolyzed and extracted as described (2, 20). In brief, following the addition of internal standards (isotopically labeled oxylipins; 1 pmol each) and an antioxidant mixture containing 0.2 mg/ml butylated hydroxytoluene, 10 mM cyclooxygenase-2-inhibiting indomethacin, 10 mM soluble epoxide hydrolase inhibitor trans-4-[4-(3-adamantan-1-yl-ureido)cyclohexyloxy]benzoic acid, and 10 mM 15-lipoxygenase-inhibiting BLX3887, proteins were precipitated with 400 μl ice-cold 2-propanol. One hundred microliters of 0.6 M KOH in methanol/H_2_O (75/25) were added to the supernatant, and saponification was carried out at 60°C for 30 min. The reaction was stopped by neutralization with acetic acid, and oxylipins were extracted by solid-phase extraction using a nonpolar/strong-anion exchange polymer material (Oasis MAX, 50 mg, 3 ml, Waters, Eschborn, Germany) (2). After drying, the solid-phase extraction eluate of each extract was reconstituted in 50 μl of methanol containing secondary internal standards (1-(1-(ethylsulfonyl)piperidin-4-yl)-3-(4-(trifluoromethoxy)phenyl)urea, 12-(3-adamantan-1-yl-ureido)-dodecanoic acid, 12-oxo-phytodienoic acid, and aleuritic acid, 40 nM each) for the calculation of the recovery of internal standards (19) and measured using LC-MS/MS.

Chromatographic separation was carried out with an Agilent 1290 Infinity II system (Agilent Technologies, Waldbronn, Germany). Solvent A was a mixture of water and solvent B (95/5) containing 0.1% acetic acid, and solvent B consisted of acetonitrile, methanol, and acetic acid (800/150/1). The oxylipins were separated on a C18 reversed-phase column (Zorbax Eclipse Plus C18, 2.1 × 150 mm, particle size 1.8 μm, pore size 9.5 nm, Agilent Technologies) at 40°C with a flow rate of 0.3 ml/min and the following gradient: isocratically 21% B from 0 min to 1.0 min, linear from 21% to 26% B in 0.5 min, linear to 51% B between 1.5 min and 10 min, linear from 51% to 66% B between 10 and 19 min, linear from 66% B to 98% B between 19 and 25.1 min, isocratically 98% B till 27.6 min and re-equilibration at 21% B between 27.7 min and 31.5 min (21, 22). For detection, the LC was coupled to a Sciex 5500 QTRAP triple quadrupole mass spectrometer (AB Sciex, Darmstadt, Germany) operated in negative electrospray ionization (ESI(−)) mode. The instrument was operated in scheduled multiple reaction monitoring, with a ±22 s window around the expected retention time and a cycle time of 0.4 min. The ion source settings were as follows: ionspray voltage: −4,500 V, nebulizer gas (gas 1, zero air): 30 psi, drying gas (gas 2, zero air): 70 psi, temperature: 650°C, and curtain gas (N_2_): 50 psi. The collision gas (N_2_) was set to 12 psi. The injection volume was 5 μl. All the details about the LC-MS/MS method according to the internationally agreed recommendations for oxylipin analysis (7) are listed in the supplemental data.

Additionally, the NIST plasma was analyzed in an independent laboratory using the same hydrolysis protocol but a different sample preparation strategy using Oasis HLB cartridges (Waters, Milford, MA) and quantified on a Sciex 6500 QTRAP equipped with a Shimadzu Nexera X2 UHPLC (Shimadzu Corporation, Japan) using a Waters 2.1 × 150 mm Acquity BEH C18 column (11, 23, 24) as described in the supplemental data (supplemental Table S1). Concentrations were calculated based on the levels of the same external calibration based on commercial standards with validated concentrations as described by Hartung *et al.* (25).

## Results

Levels of total oxylipins in NIST SRM 1950 plasma are shown in Table 1. Among the 146 oxylipins analyzed, 74 could be quantified, 70 could not be detected, and two cannot be quantified due to isobaric interferences (Table 1). Total hydroxy-PUFA from EPA and DHA occurred in low nanomolar concentrations; higher concentrations up to 70 nM were observed for arachidonic acid (ARA)-derived oxylipins and 200–400 nM for linoleic acid (LA)-derived ones. Epoxy-PUFAs were found in a similar range: 2–3 nM, <1 nM, and 15–20 nM for DHA-, EPA-, and ARA-derived ones, respectively. Again, the concentration of LA-derived EpOME was far higher between 70 and 100 nM. The concentration of dihydroxy-PUFA was about 2–10 fold lower than that of the corresponding epoxy-PUFA.

**Table 1:** Levels of oxylipins following lipid saponification (total oxylipins) in pools of human plasma (nmol/L); mean ± SD

Oxylipin	NIST SRM for human plasma (SRM 1950) (n = 4)	Pool of plasma of healthy subjects prepared under controlled conditions^a^
Isoprostanes		
15-F_1t_-IsoP (8-iso-PGF_1α_)	<1.3	<5
5-F_2c_-IsoP (8,12-iso-iPF_2α_-VI)	<2.5	<5
5-F_2t_-IsoP (5-iPF_2α_-VI)	0.74 ± 0.16	<1.3
15-F_2t_-IsoP (8-iso-PGF_2a_)	<1.3	<1.3
2,3-dinor-15-F_2t_-IsoP	<0.25	<0.38
15-oxo-15-F_2t_-IsoP	<1.3	<2.5
13,14-dihydro-15-oxo-15-F_2t_-IsoP	<5	<5
15-F_3t_-IsoP (8-iso-PGF_3α_)	<25	<25
Prostaglandins		
PGB_1_	<0.25	Not reported
PGF_1α_	<0.38	<0.5
PGB_2_	<1.3	<0.5
PGJ_2_	<0.14	<0.14
Δ12-PGJ_2_	<0.43	Not reported
15-deoxy-Δ12,14-PGJ_2_	<0.50	Not reported
6-keto-PGF_1α_	<3.2	<6.4
PGF_2α_	Not reported^b^	10 ± 2
20-OH PGF_2α_	<0.80	Not reported
11β-PGF_2α_	<0.38	Not reported
2,3-dinor-11β-PGF_2α_	<0.98	Not reported
13,14-dihydro-PGF_2α_	<0.25	Not reported
15-keto-PGF_2α_	<2.5	Not reported
13,14-dihydro-15-keto-PGF_2α_	<0.50	Not reported
11β-13,14-dihydro-15-keto-PGF_2α_	<4.5	Not reported
PGB_3_	<1.3	<2.5
Δ^17^-6-keto-PGF_1α_	<2.5	<2.5
PGF_3α_	<10	<5
1a,1b-dihomo-PGF_2α_	<1.3	<2.5
Multiple hydroxylated-FA		
Leukotriene B3	<1.3	<1.25
5,12-DiHETE	Not reported^b^	0.5 ± 0.2
5,15-DiHETE	2.2 ± 0.3	<0.5
8,15-DiHETE	11.6 ± 0.3	<2.5
Leukotriene B_4_ (LTB_4_)	<0.38	<0.38
6-*trans*-LTB_4_	0.8 ± 0.1	<0.38
6-*trans*-12-epi-LTB_4_	1.5 ± 0.2	<0.5
5 (*S*),6 (*R*)-DiHETE (ARA)	<0.20	<0.20
5 (*S*),6 (*S*)-DiHETE (ARA)	<0.23	<0.22
20-OH-LTB_4_	<0.25	<0.38
20-COOH-LTB_4_	<0.83	<0.83
18-COOH-dinor-LTB_4_	<10	<13
5,6,15-TriHETE (LxA_4_)	<0.5	<1.3
5,6,15-TriHETE (6 (*S*)-LxA_4_)	<0.25	Not reported
5,14,15-TriHETE (LxB_4_)	<1.25	Not reported
Leukotriene B_5_	<0.25	Not reported
5,6,15-TriHEPE (LxA_5_)	<0.50	Not reported
5,12,18-TriHEPE (RvE_1_)	<0.25	Not reported
5,18-DiHEPE (RvE_2_)	<0.69	Not reported
5,15-DiHEPE (RvE_4_)	<0.38	Not reported
10,17-DiHDHA (PDx)	0.8 ± 0.1	<0.38
10,17-DiHDHA (NPD_1_)	<1.1	Not reported
7,14-DiHDHA (MaR1)	<2.5	Not reported
7,14-DiHDHA (7-*epi*-MaR1)	<0.50	Not reported
13,14-DiHDHA (MaR2)	<0.5	<0.38
7,8,17-TriHDHA (RvD_1_)	<0.25	Not reported
7,16,17-TriHDHA (RvD_2_)	<2.5	Not reported
4,11,17-TriHDHA (RvD_3_)	<0.38	Not reported
4,5,17-TriHDHA (RvD_4_)	<0.5	<1.3
7,17-DiHDHA (RvD_5_)	<1.25	<1.3
Trihydroxylated α-linolenic acid and LA		
9,10,11-TriHOME	<0.25	0.7 ± 0.2
9,10,13-TriHOME	2.42 ± 0.07	1.7 ± 0.4
9,12,13-TriHOME	6.0 ± 0.4	5.3 ± 1.3
9,10,11-TriHODE	<0.25	<0.30
9,10,13-TriHODE	<2.5	<5
9,12,13-TriHODE	<0.25	<0.38
Hydroxy-FA		
9-HODE	264 ± 9	62 ± 5
10-HODE	10.1 ± 0.4	3.8 ± 0.1
12-HODE	6.8 ± 0.6	2.0 ± 0.1
13-HODE	395 ± 14	82 ± 7
15-HODE	6.7 ± 0.2	5.6 ± 0.1
9-HOTrE	6.6 ± 0.5	2.5 ± 0.1
13-HOTrE	11.3 ± 1.1	<5
13-γ-HOTrE	9.6 ± 0.7	<5
5-HETrE	2.4 ± 0.2	2.0 ± 0.3
8-HETrE	9.7 ± 0.5	3.7 ± 0.3
12-HETrE	11.3 ± 0.5	3.9 ± 0.4
15-HETrE	11.6 ± 0.7	3.8 ± 0.4
5-HETE	49 ± 3	13.9 ± 0.4
8-HETE	45 ± 4	12.8 ± 0.7
9-HETE	78 ± 5	17 ± 1
11-HETE	70 ± 6	19.1 ± 0.8
12-HETE	70 ± 4	18 ± 1
15-HETE	68 ± 4	7.6 ± 0.2
16-HETE	1.3 ± 0.1	0.78 ± 0.03
17-HETE	<0.38	<0.38
18-HETE	0.86 ± 0.07	0.6 ± 0.1
19-HETE	<5	<13
20-HETE	3.1 ± 0.5	<2.5
12-HHTrE	<0.38	<5
Tetranor-12-HETE	0.81 ± 0.08	0.24 ± 0.01
5-HEPE	2.5 ± 0.2	1.50 ± 0.02
8-HEPE	1.8 ± 0.1	0.71 ± 0.03
9-HEPE	4.4 ± 0.1	2.4 ± 0.1
11-HEPE	1.9 ± 0.1	1.26 ± 0.02
12-HEPE	4.8 ± 0.3	2.57 ± 0.05
15-HEPE	3.7 ± 0.3	1.99 ± 0.04
18-HEPE	4.9 ± 0.4	3.2 ± 0.1
4-HDHA	5.0 ± 0.1	6.5 ± 0.3
7-HDHA	4.4 ± 0.5	4.7 ± 0.2
8-HDHA	5.4 ± 0.3	3.9 ± 0.1
10-HDHA	5.2 ± 0.2	4.5 ± 0.3
11-HDHA	7.9 ± 0.4	5.8 ± 0.1
13-HDHA	5.7 ± 0.4	4.8 ± 0.3
14-HDHA	7.4 ± 0.6	6.1 ± 0.3
16-HDHA	5.7 ± 0.4	4.1 ± 0.2
17-HDHA	6.7 ± 0.4	<8.5
20-HDHA	8.8 ± 0.4	7.6 ± 0.2
22-HDHA	<0.50	Not reported
Epoxy-FA		
9 (10)-Ep-stearic acid	174 ± 16	171 ± 22
9 (10)-EpOME	96 ± 13	145 ± 26
12 (13)-EpOME	74 ± 9	116 ± 14
9 (10)-EpODE	3.5 ± 0.3	5.3 ± 1.1
12 (13)-EpODE	1.7 ± 0.2	2.6 ± 0.5
15 (16)-EpODE	21.2 ± 1.5	21 ± 2
14 (15)-EpEDE	3.1 ± 0.4	7.3 ± 1.3
8 (9)-EpETrE	15.0 ± 1.4	29 ± 5
11 (12)-EpETrE	13.8 ± 1.5	39 ± 8
14 (15)-EpETrE	20.5 ± 2.5	57 ± 11
8 (9)-EpETE	<1.3	6 ± 2
11 (12)-EpETE	<0.38	6 ± 2
14 (15)-EpETE	<0.5	7 ± 2
17 (18)-EpETE	<1.3	12 ± 3
7 (8)-EpDPE	2.2 ± 0.1	8.9 ± 1.6
10 (11)-EpDPE	2.1 ± 0.2	11 ± 2
13 (14)-EpDPE	1.7 ± 0.3	10 ± 2
16 (17)-EpDPE	1.7 ± 0.2	10 ± 2
19 (20)-EpDPE	3.3 ± 0.3	16 ± 3
Vicinal dihydroxy-FA		
9,10-DiH-stearic acid	68 ± 4	Not reported
9,10-DiHOME	26 ± 1	18.7 ± 0.9
12,13-DiHOME	7.7 ± 0.2	9.0 ± 0.5
9,10-DiHODE	1.6 ± 0.1	0.43 ± 0.03
12,13-DiHODE	<1.25	<0.38
15,16-DiHODE	13.5 ± 0.6	13.9 ± 0.5
5,6-DiHETrE	18.1 ± 0.6	16.1 ± 0.3
8,9-DiHETrE	4.1 ± 0.2	3.6 ± 0.1
11,12-DiHETrE	1.4 ± 0.1	<0.16
14,15-DiHETrE	1.0 ± 0.1	0.74 ± 0.03
5,6-DiHETE	<10	1.9 ± 0.1
8,9-DiHETE	<0.38	0.37 ± 0.01
11,12-DiHETE	<0.25	<0.25
14,15-DiHETE	<0.25	<0.25
17,18-DiHETE	<0.6	<0.55
7,8-DiHDPE	<2.5	3.3 ± 0.1
10,11-DiHDPE	0.38 ± 0.02	0.57 ± 0.02
13,14-DiHDPE	<0.25	0.37 ± 0.02
16,17-DiHDPE	<0.38	0.46 ± 0.02
19,20-DiHDPE	1.6 ± 0.1	2.0 ± 0.1
Other		
20-COOH-ARA	10.4 ± 1.1	8.0 ± 1.3

DiHDPE, dihydroxydocosapentaenoic acid; DiHETE, dihydroxyeicosatetraenoic acid; DiHETrE, dihydroxyeicosatrienoic acid; DiHODE, dihydroxyoctadecadienoic acid; DiHOME, dihydroxyoctadecenoic acid; EpDPE, epoxydocosapentaenoic acid; EpEDE, epoxyeicosadienoic acid; EpETE, epoxyeicosatetraenoic acid; EpETrE, epoxyeicosatrienoic acid; EpODE, epoxyoctadecadienoic acid; EpOME, epoxyoctadecenoic acid; HDHA, hydroxydocosahexaenoic acid; HEPE, hydroxyeicosapentaenoic acid; HETE, hydroxyeicosatetraenoic acid HODE, hydroxyoctadecadienoic acid; HOTrE, hydroxyoctadecatrienoic acid; IsoP, isoprostane; LT, leukotriene; Lx, lipoxine; Mar, maresin; PG, prostaglandin; Rv, resolvin; TriHODE, trihydroxyoctadecadienoic acid; TriHOME, trihydroxyoctadecenoic acid.

a From Mainka *et al.* (2020) *J. Lipid Res.* (2020) 61, 1424–1436.^20^

b No quantitation possible because of coeluting interference.

Multiple hydroxylated PUFAs, including so-called specialized proresolving mediators, were not detected, with the exception of nonenzymatically formed leukotriene B_4_ isomers (6-*trans*-leukotriene B_4_ and 6-*trans*-12-epi-leukotriene B_4_), 10,17-DiHDHA (PDx), and 5,15-DiHETE with concentrations around 2 nM. 8,15-DiHETE was found at a concentration of about 10 nM.

Isoprostanes, common oxidative stress markers, were below the lower limit of quantification, and only 5-F_2t_-IsoP (5-iPF_2α_-VI) could be detected in a concentration of about 1 nM. Prostaglandins carrying a β-hydroxy-keto functionality, such as D- and E-prostaglandins, degrade under alkaline conditions (1, 2, 26, 27). Thromboxane structures are in our hands also unstable in alkaline conditions (2, 28), whereas though other studies describe it as stable (1). Consequently, reporting these compounds after alkaline hydrolysis of the lipids in the sample is not possible. The concentration of PGB_2_, the degradation product of PGE_2_ (2) and other PGs was also below the lower limit of quantification (Table 1).

We also observed poor recovery (<40%) for isotopically labeled internal standards of keto-fatty acids (d_7_-oxo-eicosatetraenoic acid and d_3_-oxo-octadecadienoic acid), indicating a degradation of these oxylipins. Thus, no concentrations of oxo-fatty acids were calculated. Two LA-derived TriHOME isomers and 20-COOH-ARA were detected in a concentration in the low nanomolar concentration range (2–10 nM).

The determined concentrations in NIST plasma were, for most oxylipins, the same (100 ± 60%) when determined in an independent laboratory using the same hydrolysis protocol but an independent analysis method (Supplemental Table S1). Only for epoxy-PUFA and 8,15-DiHETE, the independent laboratory found slightly higher (∼2-fold) levels.

Despite the storage and possible freeze–thaw cycles of the NIST SRM 1950 plasma, the detected oxylipins and concentrations determined in the NIST SRM 1950 plasma were consistent with those of freshly prepared EDTA plasma of healthy subjects prepared in our laboratory (Table 1; pool B1 in (20)). This was freshly prepared according to Koch *et al.* (19), immediately stored at −80°C, and analyzed within a few days using an antioxidant cocktail during sample preparation to prevent autoxidation. Compared to this plasma, slightly higher levels of hydroxy-PUFA and lower concentrations of epoxy-PUFA were found in NIST SRM 1950 plasma, whereas the concentrations of dihydroxy-PUFA were remarkably identical. The concentrations of total oxylipins reported here are in the same range as studies reporting the oxylipin levels in human plasma prepared under controlled conditions (5, 6, 19, 20, 29). However, detected and reported concentrations of hydroxy-PUFA in commercially obtained human plasma, including the Cayman MaxSpec Reference Plasma, are up to 100–1,000 times higher (Supplemental Table S2) (1, 20, 30).

## Discussion

The concentration of total oxylipins in NIST SRM 1950 plasma is determined using an established method for the quantification of total oxylipins in two independent laboratories (2, 19, 20). Because these levels are in the same range as described in other studies, as shown for fresh EDTA plasma (Table 1), this allows us to begin to define the expected concentration range of total oxylipins in fasting normolipidemic plasma. For different plasma pools of healthy subjects, the deviations of total concentration of the individual oxylipins were generally lower than a factor of 5 but always in the same order of magnitude (factor of 10) as reported here for the NIST plasma.

Of course, interindividual differences lead to differences in the total oxylipin plasma concentrations (17). This is best investigated for the nutritional status with n3-PUFA. Here, a diet rich in DHA and EPA increases the DHA- and EPA-derived free and total oxylipins by a factor of 3–4 (5, 15, 31). However, the concentration of the oxylipins remains within the range of the concentrations reported here for the NIST plasma.

Inappropriate storage of plasma leads to (aut)oxidation of fatty acids in the lipids (32). Thus, high levels of oxidized phospholipids (3) bearing hydroxy-PUFA and thus both, free and esterified oxylipins (1, 12, 30), are found in all commercially available plasmas. This indicates that they were not constantly stored at −80°C, required to prevent artificial oxylipin formation (19, 33); indeed, some are stored and shipped at −20°C or even in a nonfrozen state.

Dramatic high concentrations of total (multiple)-hydroxy-PUFA and epoxy-PUFA 100–1,000-fold above the concentration range reported here are found in these commercial pools of human plasma (Supplemental Table S2; (1, 20)) and also in the samples of cohort studies such as the Polish branch of the Prospective Urban and Rural Epidemiological cohort (34). Other cohort studies, such as the French Nutrinet-Santé cohort study, describe total oxylipin plasma levels in the range reported here (34).

Oxylipins are delicate analytes that can degrade and be artificially formed during sample preparation and storage. In order to understand their modulation by physiological processes, disease, or nutrition, data on the concentrations expected in the plasma of healthy human subjects are indispensable. Here, we describe such a concentration range and demonstrate that the NIST SRM 1950 is a suitable reference material for total oxylipins in human plasma.

## Data availability

All data are contained within the article or supplemental data.

## Supplemental data

This article contains supplemental data (1, 2, 19, 20, 30, 35).

## Conflict of interest

The authors declare that they have no conflicts of interest with the contents of this article.
