# Supplementary material for: Total oxylipin concentrations in NIST SRM 1950 plasma compared to fresh and commercially obtained human plasma
Source: J Lipid Res. 2026 Mar 2;67(4):101011. doi: 10.1016/j.jlr.2026.101011 (PMC13068541; doi:10.1016/j.jlr.2026.101011)
Supplement: Supplemental Tables and Information [file mmc3.pdf]

Total oxylipin concentrations in NIST SRM 1950 plasma compared to fresh and commercially obtained human plasma

Supplemental Data: LC-MS method details.

*corresponding author: Nils Helge Schebb, Food Chemistry, School of Mathematics and Natural Sciences, University of Wuppertal, Gausstrasse 20, 42119 Wuppertal, Germany; Email: nils@schebb-web.de; Phone: +49-202-439-3457

Table S3: Liquid chromatography

LC system	Agilent Infinity II (Agilent Technologies, Waldbronn, Germany)
Autosampler	Agilent 1290 Multisampler (G7167B)
Pump	Agilent 1290 High Speed Pump (G7120A)
Separation conditions	Reversed-phase
Column	Zorbax Eclipse Plus C18 (2.1 x 150 mm, 1.8 µm, 9.5 nm) (Agilent Technologies)
Guard column	Guard column (AJ0-8782) UHPLC C18 (2.1 mm ID) (Phenomenex, Aschaffenburg, Germany)
Column oven temperature	40 °C
Solvents	A: water/B 95/5 (v/v) + 0.1% acetic acid<br>B: methanol/acetonitrile/acetic acid 800/150/1 (v/v/v)
Flow rate	300 µL/min
Gradient	**Time [min]** / **%B**
	0.0 / 22
	1.0 / 22
	1.5 / 32
	10.0 / 52
	19.0 / 68
	25.1 / 98
	27.6 / 98
	27.7 / 22
	31.1 / 22

Table S4: Mass spectrometry: instrument and settings

**Instrument**	
Mass Spectrometer	Sciex QTRAP 5500 (AB Sciex, Darmstadt, Germany)
Ionization mode	ESI(-)
**Source parameters**	
Curtain gas (CUR)	50 psi
Collision gas (CAD)	high (12)
Ion spray voltage (IS)	-4500 kV
Temperature (TEM)	650°C
Ion source gas 1 (GS1)	30 psi
Ion source gas 2 (GS2)	70 psi
**MS settings**	
Operating mode	scheduled selected reaction monitoring
MRM detection window	45 sec
Cycle time	0.4 sec
Data points per peak	14–18
**Software and libraries**	
Analyst 1.6.3. (AB Sciex)	

Table S5: MS transitions and MS settings

Oxylipins were identified in samples based on (relative) retention time and MRM transition. If more than one MRM transition was monitored, also the ion ratio was taken into account. (1, 5, 6)

oxylipin	internal standard	Q1	Q3	retention time [min]	DP	EP	CE	CXP
15-F_{1t}-IsoP (8-iso-$PGF_{1\alpha}$)	d4-PGF2α	355.2	211.0	6.92	-100	-10	-35	-8
5-F_{2c}-IsoP (8,12-iso-$iPF_{2\alpha}$-VI)	d11-8,12-iso-iPF2α-VI	353.2	219.2	9.50	-90	-10	-30	-8
5-F_{2t}-IsoP (5-$iPF_{2\alpha}$-VI)	d4-8-iso-PGF2α	353.2	114.8	7.44	-85	-10	-27	-8
15-F_{2t}-IsoP (8-iso-PGF_{2a})	d4-8-iso-PGF2α	353.1	193.1	6.96	-95	-10	-34	-8
2,3-dinor-15-F_{2t}-IsoP	d4-8-iso-PGF2α	325.2	237.0	5.11	-70	-10	-18	-8
15-oxo-15-F_{2t}-IsoP	d4-8-iso-PGF2α	351.2	219.0	7.57	-75	-10	-23	-8
13,14-dihydro-15-oxo-15-F_{2t}-IsoP	d4-PGF2α	353.2	291.1	8.19	-110	-10	-30	-8
15-F_{3t}-IsoP (8-iso-$PGF_{3\alpha}$)	d4-8-iso-PGF2α	351.1	193.0	5.94	-95	-10	-27	-8
PGB_1	d4-15-deoxy-Δ12,14-PGJ2	335.4	221.0	12.38	-85	-10	-28	-7
$PGF_{1\alpha}$	d4-PGF2α	355.4	293.2	7.99	-110	-10	-35	-6
PGB_2	d4-PGB2	333.3	175.1	11.57	-80	-10	-27	-8
PGJ_2	d4-PGB2	333.3	189.2	11.48	-80	-10	-24	-8
Δ12-PGJ_2	d4-15-deoxy-Δ12,14-PGJ2	333.3	189.2	11.57	-70	-10	-21	-8
15-deoxy-Δ12,14-PGJ_2	d4-15-deoxy-Δ12,14-PGJ2	315.2	203.1	17.84	-90	-10	-28	-7
6-keto-$PGF_{1\alpha}$	d4-6-keto PGF1α	369.3	163.2	5.72	-90	-10	-35	-6
$PGF_{2\alpha}$	d4-PGF2α	353.2	193.0	7.95	-80	-10	-33	-7
20-OH $PGF_{2\alpha}$	d4-PGE2	369.3	193.0	3.62	-70	-10	-37	-7
11β-$PGF_{2\alpha}$	d4-PGE2	353.3	193.1	7.24	-70	-10	-35	-12
2,3-dinor-11β-$PGF_{2\alpha}$	d4-PGE2	325.3	163.0	6.00	-65	-10	-19	-7
13,14-dihydro-$PGF_{2\alpha}$	d4-13,14-dihydro-15-keto-PGE2	355.4	193.0	9.62	-90	-10	-34	-7
15-keto-$PGF_{2\alpha}$	d4-PGE2	351.2	219.1	9.23	-60	-10	-23	-7
13,14-dihydro-15-keto-$PGF_{2\alpha}$	d4-13,14-dihydro-15-keto-PGE2	353.3	183.3	9.77	-100	-10	-35	-10
11β-13,14-dihydro-15-keto-$PGF_{2\alpha}$	d4-13,14-dihydro-15-keto-PGE2	353.4	195.0	9.98	-85	-10	-34	-7
PGB_3	d4-PGB2	331.2	223.0	9.87	-75	-10	-24	-8
Δ^{17}-6-keto-$PGF_{1\alpha}$	d4-6-keto PGF1α	367.2	163.2	4.83	-60	-10	-35	-10
$PGF_{3\alpha}$	d4-PGF2α	351.2	193.2	6.75	-95	-10	-30	-7
1a,1b-dihomo-$PGF_{2\alpha}$	d4-PGB2	381.4	221.1	10.45	-80	-10	-37	-10
Leukotriene B_3	d11-11,12-DiHETrE	337.2	195.2	15.72	-80	-10	-21	-8
5,12-DiHETE	d4-LTB4	335.2	195.1	14.14	-80	-10	-21	-8
5,15-DiHETE	d4-LTB4	335.3	173.2	13.11	-80	-10	-20	-8
8,15-DiHETE	d4-LTB4	335.2	235.2	12.67	-90	-10	-21	-4
Leukotriene B_4 (LTB_4)	d4-LTB4	335.2	195.1	13.56	-80	-10	-22	-9
6-*trans*-LTB_4	d4-LTB4	335.2	195.1	13.08	-80	-10	-22	-9
6-*trans*-12-epi-LTB_4	d4-LTB4	335.2	195.1	13.24	-85	-10	-20	-9
5(*S*),6(*R*)-DiHETE (ARA)	d11-11,12-DiHETrE	335.2	115.1	17.00	-70	-10	-20	-8
5(*S*),6(*S*)-DiHETE (ARA)	d11-11,12-DiHETrE	335.2	115.1	17.46	-70	-10	-20	-8
20-OH-LTB_4	d4-8-iso-PGF2α	351.2	195.2	6.01	-100	-10	-24	-8
20-COOH-LTB_4	d4-8-iso-PGF2α	365.2	347.2	5.79	-100	-10	-24	-8
18-COOH-dinor-LTB_4	d4-8-iso-PGF2α	337.2	195.2	4.27	-85	-10	-24	-9
5,6,15-TriHETE (LxA_4)	d5-LxA4	351.2	235.1	9.60	-70	-10	-18	-15
		351.2	115.1	9.60	-70	-10	-19	-7
5,6,15-TriHETE (6(*S*)-LxA_4)	d5-LxA4	351.2	235.1	10.00	-70	-10	-18	-5
		351.2	115.1	10.00	-70	-10	-17	-7
5,14,15-TriHETE (LxB_4)	d5-LxA4	351.2	221.0	8.57	-70	-10	-21	-13

		351.2	233.1	8.57	-70	-10	-20	-13
Leukotriene B_5	d4-LTB4	333.3	195.2	11.58	-80	-10	-21	-8
5,6,15-TriHEPE (LxA_5)	d5-LxA4	349.1	215.0	8.15	-70	-10	-24	-13
		349.1	114.9	8.15	-70	-10	-18	-5
5,12,18-TriHEPE (RvE_1)	d5-RvD2	349.3	195.0	5.76	-80	-10	-22	-10
		349.3	161.0	5.76	-80	-10	-24	-8
5,18-DiHEPE (RvE_2)	d4-LTB4	333.2	253.3	10.86	-80	-10	-19	-9
		333.2	159.2	10.86	-80	-10	-24	-10
5,15-DiHEPE (RvE_4)	d4-LTB4	333.2	115.0	11.95	-80	-10	-19	-7
		333.2	173.0	11.95	-80	-10	-19	-7
10,17-DiHDHA (PDx)	d4-LTB4	359.1	153.1	13.29	-80	-10	-21	-10
		359.1	206.1	13.29	-80	-10	-21	-12
10,17-DiHDHA (NPD_1)	d4-LTB4	359.0	153.1	13.04	-80	-10	-21	-8
		359.0	206.1	13.04	-80	-10	-18	-12
7,14-DiHDHA (MaR1)	d4-LTB4	359.1	250.2	13.37	-80	-10	-19	-14
		359.1	221.0	13.37	-80	-10	-18	-8
7,14-DiHDHA (7-*epi*-MaR1)	d4-LTB4	359.1	250.1	12.81	-80	-10	-20	-5
		359.1	221.0	12.81	-80	-10	-19	-8
13,14-DiHDHA (MaR2)	d4-9,10-DiHOME	359.1	221.0	14.91	-70	-10	-16	-14
		359.1	167.0	14.91	-70	-10	-18	-14
7,8,17-TriHDHA (RvD_1)	d5-RvD1	375.3	141.0	9.74	-70	-10	-19	-8
		375.3	215.0	9.74	-70	-10	-24	-13
7,16,17-TriHDHA (RvD_2)	d5-RvD2	375.3	141.0	8.91	-80	-10	-21	-8
		375.3	175.0	8.91	-80	-10	-28	-12
4,11,17-TriHDHA (RvD_3)	d5-RvD2	375.3	147.0	8.59	-80	-10	-24	-10
		375.3	137.0	8.59	-80	-10	-26	-8
4,5,17-TriHDHA (RvD_4)	d5-RvD1	375.2	131.0	11.02	-80	-10	-19	-10
		375.2	259.0	11.02	-80	-10	-20	-10
7,17-DiHDHA (RvD_5)	d4-LTB4	359.1	199.1	13.36	-70	-10	-19	-10
		359.1	141.0	13.36	-70	-10	-19	-16
9,10,11-TriHOME	d5-LxA4	329.1	201.1	9.13	-95	-10	-30	-8
9,10,13-TriHOME	d5-LxA4	329.2	171.1	7.88	-100	-10	-31	-8
9,12,13-TriHOME	d5-LxA4	329.2	211.1	7.73	-100	-10	-31	-10
9,10,11-TriHODE	d5-LxA4	327.0	171.0	7.71	-80	-10	-25	-8
9,10,13-TriHODE	d5-LxA4	327.2	201.0	6.75	-80	-10	-28	-8
9,12,13-TriHODE	d5-LxA4	327.2	211.0	6.75	-80	-10	-29	-10
9-HODE	d4-9-HODE	295.2	171.1	19.19	-100	-10	-24	-7
10-HODE	d4-9-HODE	295.1	183.0	19.11	-90	-10	-25	-7
12-HODE	d4-13-HODE	295.1	183.0	18.76	-80	-10	-24	-9
13-HODE	d4-9-HODE	295.2	195.2	19.08	-100	-10	-24	-9
15-HODE	d4-13-HODE	295.2	223.0	17.94	-95	-10	-24	-9
9-HOTrE	d4-13-HODE	293.2	171.2	16.66	-85	-10	-20	-8
13-HOTrE	d4-13-HODE	293.2	195.1	17.03	-90	-10	-22	-8
13-gamma-HOTrE	d4-LTB4	293.0	193.0	17.39	-90	-10	-23	-8
5-HETrE	d8-5-HETE	321.2	115.1	23.63	-90	-10	-17	-9
8-HETrE	d8-5-HETE	321.2	157.1	21.70	-85	-10	-22	-9
12-HETrE	d8-5-HETE	321.0	181.0	21.93	-85	-10	-24	-10
15-HETrE	d8-5-HETE	321.2	221.2	21.29	-90	-10	-21	-10
5-HETE	d8-5-HETE	319.2	115.2	21.52	-80	-10	-19	-7
8-HETE	d8-12-HETE	319.2	155.2	20.81	-80	-10	-20	-6
9-HETE	d8-5-HETE	319.2	167.2	21.21	-80	-10	-21	-7
11-HETE	d8-12-HETE	319.2	167.2	20.41	-80	-10	-21	-7
12-HETE	d8-12-HETE	319.2	179.2	20.86	-80	-10	-19	-8
15-HETE	d8-15-HETE	319.2	219.2	19.84	-80	-10	-18	-8
16-HETE	d8-15-HETE	319.2	233.1	18.66	-85	-10	-18	-8

17-HETE	d8-15-HETE	319.2	247.0	18.50	-85	-10	-19	-8
18-HETE	d8-15-HETE	319.2	261.0	18.28	-85	-10	-20	-8
19-HETE	d8-15-HETE	319.3	230.9	17.57	-70	-10	-19	-6
20-HETE	d6-20-HETE	319.2	289.1	17.84	-100	-10	-22	-6
12-HHTrE	d11-11,12-DiHETrE	279.1	179.0	15.40	-70	-10	-15	-8
tetranor-12-HETE	d4-9,10-DiHOME	265.2	109.1	14.86	-70	-10	-15	-8
5-HEPE	d8-15-HETE	317.2	115.1	18.88	-80	-10	-18	-6
8-HEPE	d8-15-HETE	317.2	155.2	18.18	-80	-10	-18	-8
9-HEPE	d8-15-HETE	317.2	167.0	18.54	-70	-10	-17	-8
11-HEPE	d8-15-HETE	317.2	167.0	17.97	-70	-10	-19	-8
12-HEPE	d8-15-HETE	317.2	179.2	18.38	-85	-10	-18	-8
15-HEPE	d8-15-HETE	317.2	219.2	17.85	-80	-10	-18	-10
18-HEPE	d8-15-HETE	317.2	259.2	17.11	-75	-10	-15	-7
4-HDHA	d8-5-HETE	343.2	101.1	22.12	-75	-10	-17	-7
7-HDHA	d8-5-HETE	343.2	141.2	21.12	-75	-10	-17	-7
8-HDHA	d8-5-HETE	343.2	189.2	21.36	-70	-10	-17	-7
10-HDHA	d8-12-HETE	343.2	153.2	20.63	-70	-10	-19	-7
11-HDHA	d8-5-HETE	343.2	121.1	20.97	-70	-10	-18	-7
13-HDHA	d8-12-HETE	343.2	193.2	20.36	-75	-10	-17	-7
14-HDHA	d8-12-HETE	343.2	205.2	20.63	-70	-10	-17	-7
16-HDHA	d8-15-HETE	343.2	233.2	20.05	-75	-10	-17	-7
17-HDHA	d8-15-HETE	343.2	245.1	20.18	-80	-10	-18	-6
20-HDHA	d8-15-HETE	343.2	241.2	19.50	-75	-10	-17	-7
22-HDHA	d6-20-HETE	343.2	313.2	18.93	-85	-10	-18	-7
9(10)-Ep-stearic acid	d4-12(13)-EpOME	297.0	170.8	24.03	-120	-10	-24	-11
9(10)-EpOME	d4-12(13)-EpOME	295.2	171.1	22.43	-105	-10	-19	-8
12(13)-EpOME	d4-12(13)-EpOME	295.2	195.2	22.20	-105	-10	-19	-8
9(10)-EpODE	d4-12(13)-EpOME	293.2	171.2	19.84	-90	-10	-16	-8
12(13)-EpODE	d4-12(13)-EpOME	293.2	183.1	20.30	-90	-10	-20	-8
15(16)-EpODE	d4-12(13)-EpOME	293.3	235.2	19.67	-90	-10	-16	-4
14(15)-EpEDE	d11-14(15)-EpETrE	321.2	221.2	23.59	-85	-10	-19	-4
8(9)-EpETrE	d11-8(9)-EpETrE	319.2	155.2	23.22	-90	-10	-16	-6
11(12)-EpETrE	d11-8(9)-EpETrE	319.2	167.2	23.04	-85	-10	-16	-7
14(15)-EpETrE	d11-14(15)-EpETrE	319.2	219.2	22.43	-90	-10	-15	-4
8(9)-EpETE	d11-8(9)-EpETrE	317.2	127.2	21.08	-90	-10	-16	-6
11(12)-EpETE	d11-14(15)-EpETrE	317.2	167.0	20.89	-90	-10	-16	-6
14(15)-EpETE	d11-14(15)-EpETrE	317.2	207.2	20.71	-90	-10	-16	-6
17(18)-EpETE	d11-14(15)-EpETrE	317.2	215.2	19.94	-90	-10	-16	-6
7(8)-EpDPE	d11-8(9)-EpETrE	343.2	141.2	23.16	-75	-10	-16	-7
10(11)-EpDPE	d11-8(9)-EpETrE	343.2	153.2	22.95	-90	-10	-16	-7
13(14)-EpDPE	d11-14(15)-EpETrE	343.2	193.2	22.84	-90	-10	-16	-7
16(17)-EpDPE	d11-14(15)-EpETrE	343.2	233.2	22.74	-90	-10	-16	-7
19(20)-EpDPE	d11-14(15)-EpETrE	343.2	241.2	22.21	-90	-10	-16	-7
9,10-DiH-stearic acid	d4-9,10-DiHOME	315.2	170.8	16.04	-85	-10	-35	-9
9,10-DiHOME	d4-9,10-DiHOME	313.2	201.2	14.67	-105	-10	-28	-8
12,13-DiHOME	d4-9,10-DiHOME	313.2	183.2	14.21	-105	-10	-29	-8
9,10-DiHODE	d4-9,10-DiHOME	311.2	201.2	12.52	-90	-10	-26	-10
12,13-DiHODE	d4-9,10-DiHOME	311.2	183.1	12.62	-105	-10	-29	-8
15,16-DiHODE	d4-9,10-DiHOME	311.2	223.2	12.46	-105	-10	-28	-10
5,6-DiHETrE	d11-11,12-DiHETrE	337.2	145.1	17.81	-95	-10	-25	-10
8,9-DiHETrE	d11-11,12-DiHETrE	337.2	127.1	16.88	-95	-10	-29	-8
11,12-DiHETrE	d11-11,12-DiHETrE	337.2	167.1	16.24	-90	-10	-25	-8
14,15-DiHETrE	d11-11,12-DiHETrE	337.2	207.1	15.44	-90	-10	-24	-10
5,6-DiHETE	d4-LTB4	335.2	115.1	15.26	-85	-10	-20	-8
8,9-DiHETE	d4-LTB4	335.2	127.1	14.42	-90	-10	-25	-5

11,12-DiHETE	d4-LTB4	335.2	167.1	14.04	-90	-10	-25	-5
14,15-DiHETE	d4-LTB4	335.3	207.2	13.81	-90	-10	-24	-10
17,18-DiHETE	d4-LTB4	335.3	247.2	13.18	-90	-10	-23	-8
7,8-DiHDPE	d11-11,12-DiHETrE	361.2	113.1	17.63	-90	-10	-23	-6
10,11-DiHDPE	d11-11,12-DiHETrE	361.2	153.2	16.81	-90	-10	-23	-6
13,14-DiHDPE	d11-11,12-DiHETrE	361.2	193.2	16.43	-90	-10	-23	-6
16,17-DiHDPE	d11-11,12-DiHETrE	361.2	233.2	16.15	-90	-10	-23	-6
19,20-DiHDPE	d11-11,12-DiHETrE	361.2	273.2	15.51	-90	-10	-23	-6
20-COOH-ARA	d6-20-HETE	333.2	271.0	16.91	-95	-10	-23	-6
d11-8,12-iso-iPF2α-VI		364.2	320.1	9.45	-90	-10	-30	-8
d4-13,14-dihydro-15-keto-PGE2		355.4	239.1	10.36	-65	-10	-31	-7
d4-15-deoxy-Δ12,14-PGJ2		319.4	203.0	17.84	-80	-10	-31	-7
d4-6-keto-PGF1α		373.0	167.0	5.71	-100	-10	-35	-8
d4-8-iso-PGF2α		357.2	196.8	6.94	-75	-10	-34	-8
d4-LTB4		339.2	197.2	13.50	-80	-10	-22	-9
d4-PGB2		337.2	179.0	11.54	-80	-10	-27	-8
d4-PGE2		355.2	275.3	8.30	-80	-10	-24	-6
d4-PGF2α		357.2	197.0	7.92	-80	-10	-33	-7
d5-LxA4		356.3	222.2	9.54	-80	-10	-24	-13
d5-RvD1		380.3	141.0	9.68	-80	-10	-18	-8
d5-RvD2		380.2	175.0	8.86	-80	-10	-31	-10
d4-9-HODE		299.2	172.3	19.08	-100	-10	-24	-6
d4-13-HODE		299.2	198.1	18.97	-80	-10	-24	-9
d8-5-HETE		327.2	116.1	21.36	-80	-10	-19	-8
d8-12-HETE		327.2	184.2	20.66	-85	-10	-20	-8
d8-15-HETE		327.2	226.0	19.64	-90	-10	-18	-8
d6-20-HETE		325.2	295.2	17.75	-90	-10	-22	-6
d4-9,10-DiHOME		317.2	203.4	14.57	-105	-10	-28	-8
d11-11,12-DiHETrE		348.2	167.2	16.08	-85	-10	-26	-8
d4-12(13)-EpOME		299.2	198.1	22.09	-100	-10	-21	-8
d11-8(9)-EpETrE		330.2	155.0	23.10	-80	-10	-16	-7
d11-14(15)-EpETrE		330.2	219.3	22.29	-90	-10	-16	-4

Definition of LLOQ and LOD

The limit of detection (LOD) and lower limit of quantification (LLLOQ) were determined as described (1). The LOD was set to the concentration of the lowest standard injected yielding a signal-to-noise-ratio (S/N) ≥ 3. The lower limit of quantification (LLOQ) was set to the concentration of the lowest calibration standard yielding an S/N ≥ 5 and an accuracy within 100±20% using the linear calibration function.

Table S6: LLOQ [nM, injected solution, injection volume 5 µL] (S/N≥5) of quantified oxylipins determined in matrix free standard solutions.

oxylipin	LLOQ	oxylipin	LLOQ	oxylipin	LLOQ
15-F$_{1t}$-IsoP (8-iso-PGF$_{1\alpha}$)	1.3	7,14-DiHDHA (MaR1)	2.5	10-HDHA	0.3
5-F$_{2c}$-IsoP (8,12-iso-iPF$_{2\alpha}$-VI)	2.5	7,14-DiHDHA (7-*epi*-MaR1)	0.5	11-HDHA	0.3
5-F$_{2t}$-IsoP (5-iPF$_{2\alpha}$-VI)	0.3	13,14-DiHDHA (MaR2)	0.5	13-HDHA	0.4
15-F$_{2t}$-IsoP (8-iso-PGF$_{2a}$)	1.3	7,8,17-TriHDHA (RvD$_1$)	0.3	14-HDHA	0.7
2,3-dinor-15-F$_{2t}$-IsoP	0.3	7,16,17-TriHDHA (RvD$_2$)	2.5	16-HDHA	0.3
15-oxo-15-F$_{2t}$-IsoP	1.3	4,11,17-TriHDHA (RvD$_3$)	0.4	17-HDHA	0.9
13,14-dihydro-15-oxo-15-F$_{2t}$-IsoP	5	4,5,17-TriHDHA (RvD$_4$)	0.5	20-HDHA	1.3
15-F$_{3t}$-IsoP (8-iso-PGF$_{3\alpha}$)	25	7,17-DiHDHA (RvD$_5$)	1.3	22-HDHA	0.5
PGB$_1$	0.3	9,10,11-TriHOME	0.3	9(10)-Ep-stearic acid	5
PGF$_{1\alpha}$	0.4	9,10,13-TriHOME	1.3	9(10)-EpOME	0.3
PGB$_2$	1.3	9,12,13-TriHOME	0.5	12(13)-EpOME	0.5
PGJ$_2$	0.1	9,10,11-TriHODE	0.3	9(10)-EpODE	0.3
Δ12-PGJ$_2$	0.4	9,10,13-TriHODE	2.5	12(13)-EpODE	0.5
15-deoxy-Δ12,14-PGJ$_2$	0.5	9,12,13-TriHODE	0.3	15(16)-EpODE	0.5
6-keto-PGF$_{1\alpha}$	3.2	9-HODE	0.5	14(15)-EpEDE	0.3
PGF$_{2\alpha}$	1.3	10-HODE	0.4	8(9)-EpETrE	2.5
20-OH PGF$_{2\alpha}$	0.8	12-HODE	0.3	11(12)-EpETrE	0.3
11β-PGF$_{2\alpha}$	0.4	13-HODE	2.5	14(15)-EpETrE	0.5
2,3-dinor-11β-PGF$_{2\alpha}$	1.0	15-HODE	0.9	8(9)-EpETE	1.3
13,14-dihydro-PGF$_{2\alpha}$	0.3	9-HOTrE	0.4	11(12)-EpETE	0.38
15-keto-PGF$_{2\alpha}$	2.5	13-HOTrE	3.1	14(15)-EpETE	0.5
13,14-dihydro-15-keto-PGF$_{2\alpha}$	0.5	13-gamma-HOTrE	5	17(18)-EpETE	1.3
11β-13,14-dihydro-15-keto-PGF$_{2\alpha}$	4.5	5-HETrE	0.3	7(8)-EpDPE	1.1
PGB$_3$	1.3	8-HETrE	1.3	10(11)-EpDPE	0.3
Δ^{17}-6-keto-PGF$_{1\alpha}$	2.5	12-HETrE	0.4	13(14)-EpDPE	0.4
PGF$_{3\alpha}$	10	15-HETrE	0.4	16(17)-EpDPE	0.5
1a,1b-dihomo-PGF$_{2\alpha}$	1.3	5-HETE	0.2	19(20)-EpDPE	1.3
Leukotriene B$_3$	1.3	8-HETE	1.2	9,10-DiH-stearic acid	2.5
5,12-DiHETE	0.5	9-HETE	1.3	9,10-DiHOME	0.3
5,15-DiHETE	0.5	11-HETE	0.2	12,13-DiHOME	0.2
8,15-DiHETE	2.5	12-HETE	1.3	9,10-DiHODE	0.3
Leukotriene B$_4$ (LTB$_4$)	0.4	15-HETE	0.6	12,13-DiHODE	1.3
6-*trans*-LTB$_4$	0.5	16-HETE	0.4	15,16-DiHODE	0.7
6-*trans*-12-epi-LTB$_4$	1.3	17-HETE	0.4	5,6-DiHETrE	0.3
5(*S*),6(*R*)-DiHETE (ARA)	0.2	18-HETE	0.5	8,9-DiHETrE	0.2
5(*S*),6(*S*)-DiHETE (ARA)	0.2	19-HETE	5	11,12-DiHETrE	0.2
20-OH-LTB$_4$	0.3	20-HETE	2.5	14,15-DiHETrE	0.3
20-COOH-LTB$_4$	0.8	12-HHTrE	0.4	5,6-DiHETE	10
18-COOH-dinor-LTB$_4$	10	tetranor-12-HETE	0.3	8,9-DiHETE	0.4
5,6,15-TriHETE (LxA$_4$)	0.5	5-HEPE	0.2	11,12-DiHETE	0.3
5,6,15-TriHETE (6(*S*)-LxA$_4$)	0.3	8-HEPE	0.2	14,15-DiHETE	0.3
5,14,15-TriHETE (LxB$_4$)	1.3	9-HEPE	0.5	17,18-DiHETE	0.6
Leukotriene B$_5$	0.3	11-HEPE	0.2	7,8-DiHDPE	2.5
5,6,15-TriHEPE (LxA$_5$)	0.5	12-HEPE	0.3	10,11-DiHDPE	0.3
5,12,18-TriHEPE (RvE$_1$)	0.3	15-HEPE	1.4	13,14-DiHDPE	0.3
5,18-DiHEPE (RvE$_2$)	0.7	18-HEPE	0.5	16,17-DiHDPE	0.4
5,15-DiHEPE (RvE$_4$)	0.4	4-HDHA	0.3	19,20-DiHDPE	1.3
10,17-DiHDHA (PDx)	0.6	7-HDHA	1.3	20-COOH-ARA	1.3
10,17-DiHDHA (NPD$_1$)	1.1	8-HDHA	1.0		

Sample preparation

Details on sample preparation, including an SOP can be found in (2-4). In brief, internal standards (deuterium-labeled oxylipins [each 1 pmol/100 µL plasma]) and additives (10 µL 0.2 mg/mL BHT, 100 µM indomethacin and 100 µM t-AUCB in methanol) were added to 100 µL freshly thawed plasma and samples were mixed. 400 µL ice-cold iso-propanol were added, and samples were frozen at -80°C for at least 30 min, and subsequently centrifuged (4°C, 10 min, 20 000 x g). The supernatant was hydrolyzed at 60°C for 30 min using 100 µL 0.6 M KOH in methanol/water (75/25, *v/v*). Following hydrolysis, samples were cooled and neutralized with ~20 µL 25% aqueous acetic acid followed by the transfer onto the preconditioned SPE cartridge.

Oxylipins were extracted using Oasis MAX extraction cartridges (60 mg, 3 mL; Waters, Eschborn, Germany) The SPE cartridges were preconditioned with one cartridge volume of each, ethyl acetate/*n*-hexane (75/25, *v/v*) containing 1% acetic acid, methanol and 0.1 M disodium hydrogen phosphate adjusted to pH 6.0 with acetic acid in water/MeOH (95/5, *v/v*). 2 mL 0.1 M aqueous disodium hydrogen phosphate buffer (pH 6.0) were added together with the sample onto the preconditioned cartridge. The cartridge was washed with 3 mL water and 3 mL water/methanol (50/50, *v/v*) and dried under vacuum (-200 mbar) for 30 s. The analytes were eluted with 2.0 mL of 75/25 (*v/v*) ethyl acetate/*n*-hexane with 1% acetic acid in glass tubes containing 6 µL of 30% glycerol in methanol. The solvent was evaporated using a vacuum concentrator (1 mbar, 30 °C, 45-60 min; Christ, Osterode am Harz, Germany) and samples were resuspended in 50 µL methanol containing 1-(1-(ethylsulfonyl)piperidin-4-yl)-3-(4-(trifluoromethoxy)phenyl)urea, 12-(3-adamantan-1-yl-ureido)-dodecanoic acid, 12-oxo-phytodienoic acid and aleuritic acid, 40 nM each, as secondary internal standard for the calculation of the recovery of the IS, centrifuged (10 min, 4 °C, 20,000×g) and analyzed by LC-MS.

A pool of human plasma from healthy subjects was used as QC sample, and IS recovery, accuracy and precision of the quantified oxylipins were evaluated in the sample batch.

Table S7: Recovery of internal standards in human plasma samples (NIST SRM 1950)

internal standard	recovery [%] (Mean ± SD; n=4)
d11-8,12-iso-iPF2α-VI	98±7
d4-6-keto-PGF1α	72±3
d4-8-iso-PGF2α	79±5
d4-LTB4	90±6
d4-PGB2	111±13
d4-PGF2α	79±4
d5-LxA4	75±4
d5-RvD1	74±4
d5-RvD2	78±4
d4-9-HODE	89±6
d4-13-HODE	83±8
d8-5-HETE	82±7
d8-12-HETE	87±9
d8-15-HETE	86±8
d6-20-HETE	93±11
d4-9,10-DiHOME	60±2
d11-11,12-DiHETrE	85±5
d4-12(13)-EpOME	73±7
d11-8(9)-EpETrE	77±7
d11-14(15)-EpETrE	84±8
d7-5-oxo-ETE	not recovered
d3-13-oxo-ODE	39±3

References

1. Kutzner, L., K. M. Rund, A. I. Ostermann, N. M. Hartung, J.-M. Galano, L. Balas, T. Durand, M. S. Balzer, S. David, and N. H. Schebb. 2019. Development of an Optimized LC-MS Method for the Detection of Specialized Pro-Resolving Mediators in Biological Samples. *Frontiers in Pharmacology*.

2. Mainka, M., C. Dalle, M. Pétéra, J. Dalloux-Chioccioli, N. Kampschulte, A. I. Ostermann, M. Rothe, J. Bertrand-Michel, J. W. Newman, C. Gladine, and N. H. Schebb. 2020. Harmonized procedures lead to comparable quantification of total oxylipins across laboratories. *Journal of Lipid Research* **61**: 1424-1436.

3. Koch, E., M. Mainka, C. Dalle, A. I. Ostermann, K. M. Rund, L. Kutzner, L.-F. Froehlich, J. Bertrand-Michel, C. Gladine, and N. H. Schebb. 2020. Stability of oxylipins during plasma generation and long-term storage. *Talanta* **217**: 121074.

4. Ostermann, A. I., E. Koch, K. M. Rund, L. Kutzner, M. Mainka, and N. H. Schebb. 2020. Targeting esterified oxylipins by LC–MS - Effect of sample preparation on oxylipin pattern. *Prostaglandins & Other Lipid Mediators* **146**: 106384.

5. Hartung, N. M., M. Mainka, R. Pfaff, M. Kuhn, S. Biernacki, L. Zinnert, N. H. Schebb. 2023. Development of a quantitative proteomics approach for cyclooxygenases and lipoxygenases in parallel to quantitative oxylipin analysis allowing the comprehensive investigation of the arachidonic acid cascade. *Anal BioanalChem* **415(5)**: 1-21.

6. Rund, K. M., A. I. Ostermann, L. Kutzner, J.-M. Galano, C. Oger, C. Vigor, S. Wecklein, N. Seiwert, T. Durand, N. H. Schebb. 2018. Development of an LC-(ESI-)-MS/MS method for the simultaneous quantification of 35 isoprostanes and isofurans derived from the major n3- and n6-PUFAs. *Anal Chim Acta* **1037**: 63-74.
